# Changes in seminal parameters among Brazilian men between 1995 and 2018

**DOI:** 10.1038/s41598-020-63468-9

**Published:** 2020-04-14

**Authors:** Samyra Siqueira, Anne Caroline Ropelle, Josiane A. A. Nascimento, Francisco A. T. Fazano, Luis Guillermo Bahamondes, José Roberto Gabiatti, Lúcia Costa-Paiva, Luiz Francisco Baccaro

**Affiliations:** Department of Obstetrics and Gynecology, University of Campinas (UNICAMP), Rua Alexander Fleming, 101, Campinas, 13.083-881 São Paulo Brazil

**Keywords:** Infertility, Urological manifestations

## Abstract

Aiming to investigate trends in seminal parameter values among Brazilian men between 1995 and 2018, we performed a retrospective analysis of spermograms of couples admitted for infertility testing at UNICAMP/Brazil. For the present study, only the first sample produced by each man was analyzed (9,267 samples). Total motile sperm count (TMSC), percentage of spermatozoa with normal morphology (NM), and sperm concentration after seminal processing (SCA) were considered dependent variables. Statistical analysis was carried out through linear regression for the median values both in the general population and in the population categorized by age group (<30, 30–39, and ≥40 years). During the study period, the mean age of men was 32.46 (± 6.48) years, with a median of 32 (18–67) years. We found a significant decrease in the median values of TMSC (reduction of 2.84 million/year), NM (reduction of 0.52% each year) and SCA (reduction of 0.24 million/mL each year). In conclusion, we observed that Brazilian men undergoing infertility investigation had a decline in seminal parameters in the past 23 years. Surveillance should be maintained in the coming years, and further studies are needed to elucidate possible causes for seminal deterioration and to devise strategies to reverse this trend.

## Introduction

Infertility affects over 186 million people worldwide, predominately from developing countries^1^. It is recognized by the World Health Organization (WHO) as a disease and a public health problem that can cause harmful physical and psychological consequences for both women and men, as well as psychological distress and social stigmatization^2^. The male factor accounts for 20% of infertility cases and contributes to about 30–40% of cases. Seminal analysis is a necessary step to assess possible infertility^3^.

A possible decline in world seminal parameters has been suggested by several researchers. A study published in 2017 noted a decline in seminal quality in young Chinese men over a 15-year period^4^. Over the past 35 years, an average 57% decline in sperm concentration has been reported worldwide^5^. From 1973 to 2011, authors reported a 50–60% decline in male sperm concentration in North America, Europe, Australia, and New Zealand^6^. In Brazil, some authors have also published data on declining concentrations and percentage of morphologically normal sperm^7,8^. Possible reasons for these changes in seminal parameters may be related to lifestyle, habits, age, alcohol or tobacco consumption, as well as endocrine factors, obesity, diet, and coffee consumption^9^.

Although studies suggest a decline in seminal quality in men around the world, there is still some doubt as to whether this phenomenon has actually been occurring, or whether the observed decline could be due to changes in laboratory techniques and the different statistical approaches used to study the subject^10^. Thus, this study aimed to investigate the changes in seminal parameters over the past 23 years in Brazilian men who underwent marital infertility investigation in a hospital in southeastern Brazil.

## Results

### Subjects and samples

**Figure 1:**
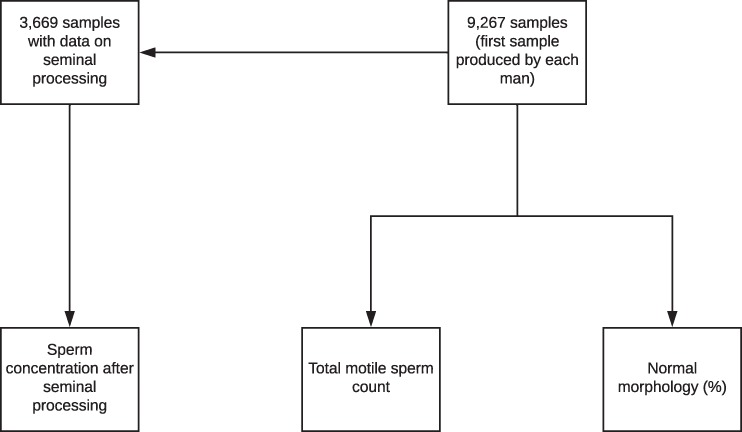
Study population between 1995 and 2018.

In total, we evaluated 9,267 samples produced by 9,267 men (1 sample per man) who underwent infertility testing between January 1995 and June 2018 (Fig. 1). During the study period, the mean age of these men was 32.46 (±6.48) years, with a median of 32.00 (range 18.00 to 67.00) years. By stratifying the men by age group, 3,166 (34.16%) men were under 30 years of age, 4,936 (53.26%) were between 30 and 39 years of age, and 1,165 (12.58%) were 40 years of age or older. The main characteristics of the samples collected throughout the study period are shown in Table 1.

**Table 1:** Men’s age at sample collection and main characteristics of the samples collected throughout the study period (9,267).

Parameter	Mean (SD)	Minimum	25%	Median	75%	Maximum
Age (years)	32.46 (6.48)	18.00	28.00	32.00	36.00	67.00
Volume (ml)	3.02 (1.57)	0.10	2.00	2.80	4.00	20.00
Sperm concentration (millions/ml)	67.26 (69.51)	0.00	14.00	45.00	100.00	400.00
Total sperm number	194.89 (226.65)	0.00	34.00	121.50	280.50	3400.00
TMSC	87.02 (113.31)	0.00	9.68	44.64	123.00	1896.0
Vitality (live spermatozoa, %)	64.33 (26.22)	0.00	63.00	73.00	80.00	100.00
Sperm morphology (normal forms, %)	8.31 (6.20)	0.00	3.00	8.00	12.00	68.00
Sperm concentration after seminal processing*	10.08 (16.54)	0.00	0.60	4.80	11.50	200.00

TMSC: total motile sperm count.

*3,669 samples.

### Total motile sperm count

**Figure 2:**
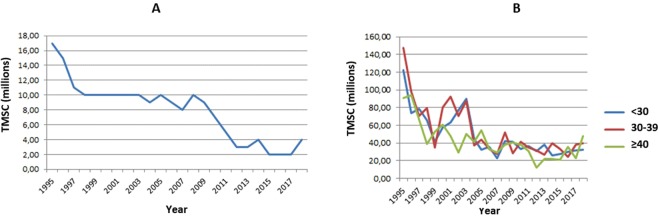
Total motile sperm counts (median) between 1995 and 2018 - linear regression (n = 9,267). TMSC – Total motile sperm count (millions). (**A**)– Total group. Total (n = 9,267). Intercept or linear coefficient: A = 82.8; SE(A) = 6.81; t = 12.16; P < 0.001. Inclination or angular coefficient: B = −2.839; SE(B) = 0.507; t = −5.60; P < 0.001. (**B**) – Population categorized by age group. Age < 30 years-old. Intercept or linear coefficient: A = 79.59; SE(A) = 6.46; t = 12.32; p < 0.001. Inclination or angular coefficient: B = −2.671; SE(B) = 0.481; t = −5.55; p < 0.001. Age 30–39 years-old. Intercept or linear coefficient: A = 90.77; SE(A) = 8.32; t = 10.91; p < 0.001. Inclination or angular coefficient: B = −3.210; SE(B) = 0.620; t = −5.18; p < 0.001. Age ≥ 40 years-old. Intercept or linear coefficient: A = 66.74; SE(A) = 5.64; t = 11.84; p < 0.001. Inclination or angular coefficient: B = −2.108; SE(B) = 0.420; t = −5.02; p < 0.001.

Regarding the total motile sperm count (TMSC) in the samples, we found a significant trend towards a decrease in median value over the years (reduction of 2.84 million progressive motile sperm each year) (Fig. 2A). Controlling for age, we observed a similar downward trend in the median TMSC in the three age groups evaluated (a reduction of 2.67 million progressive motile sperm each year in the age group <30 years, a reduction of 3.21 million progressive motile sperm each year in the age group between 30 and 39 years, and a reduction of 2.11 million progressive motile sperm each year in the age group ≥40 years) (Fig. 2B).

### Sperm morphology

**Figure 3:**
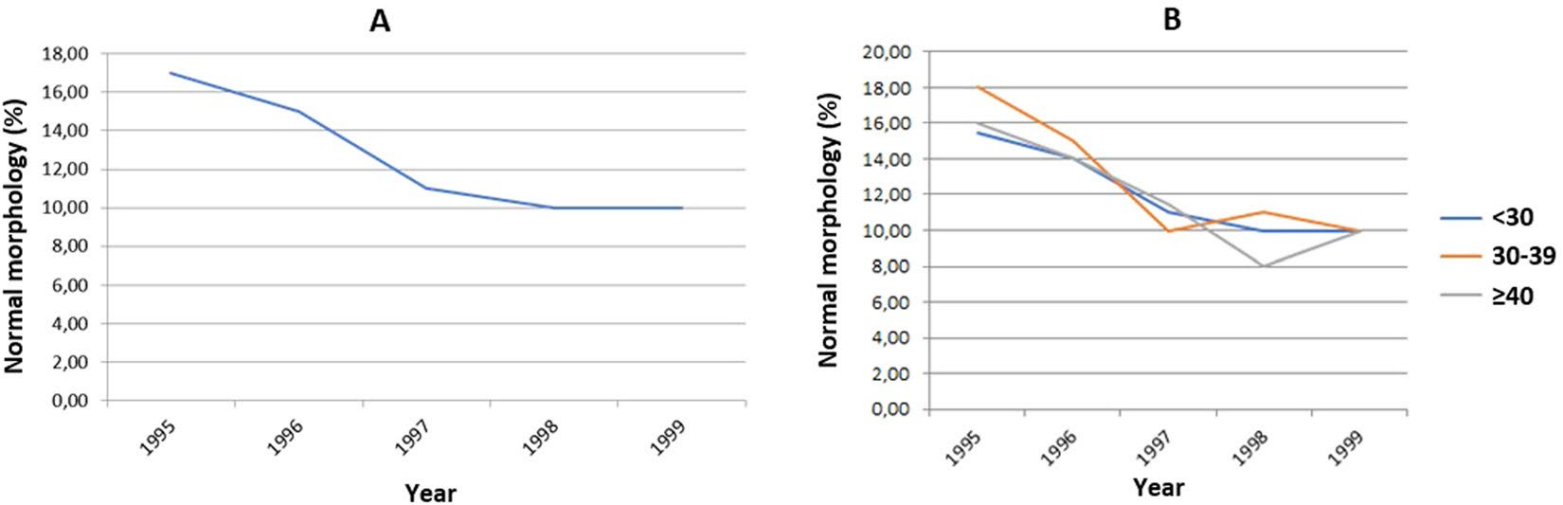
Sperm with normal morphology (median) between 1995 and 1999 - linear regression (n = 2,169). (**A**) – Total group. Total (n = 2,169). Intercept or linear coefficient: A = 16.40; SE(A) = 1.01; t = 16.24; p < 0.001. Inclination or angular coefficient: B = −1.900; SE(B) = 0.412; t = −4.61; p = 0.01. (**B**) – Population categorized by age group. Age < 30 years-old. Intercept or linear coefficient: A = 15.10; SE(A) = 0.73; t = 20.55; p < 0.001. Inclination or angular coefficient: B = −1.500; SE(B) = 0.300; t = −5.00; p = 0.01. Age 30–39 years-old. Intercept or linear coefficient: A = 16.80; SE(A) = 1.47; t = 11.43; p = 0.001. Inclination or angular coefficient: B = −2.000; SE(B) = 0.600; t = −3.33; p = 0.04. Age ≥ 40 years-old. Intercept or linear coefficient: A = 15.50; SE(A) = 1.25; t = 12.41; p < 0.001. Inclination or angular coefficient: B = −1.800; SE(B) = 0.510; t = −3.53; p = 0.03.

**Figure 4:**
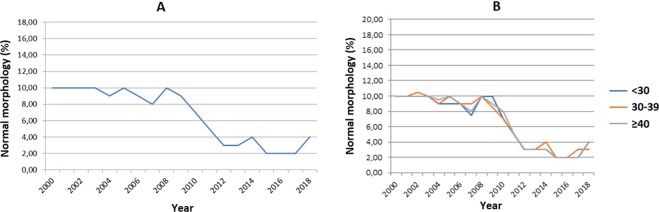
Sperm with normal morphology (median) between 2000 and 2018 - linear regression (n = 7,098). (**A**) – Total group. Total (n = 7,098). Intercept or linear coefficient: A = 11.45; SE(A) = 0.62; t = 18.32; p < 0.001. Inclination or angular coefficient: B = −0.530; SE(B) = 0.059; t = −8.93; p < 0.001. (**B**) – Population categorized by age group. Age < 30 years-old. Intercept or linear coefficient: A = 11.37; SE(A) = 0.67; t = 16.96; p < 0.001. Inclination or angular coefficient: B = −0.530; SE(B) = 0.064; t = −8.32; p < 0.001. Age 30–39 years-old. Intercept or linear coefficient: A = 11.61; SE(A) = 0.59; t = 19.73; p < 0.001. Inclination or angular coefficient: B = −0.541; SE(B) = 0.056; t = −9.69; p < 0.001. Age ≥ 40 years-old. Intercept or linear coefficient: A = 11.58; SE(A) = 0.66; t = 17.63; p < 0.001. Inclination or angular coefficient: B = −0.541; SE(B) = 0.062; t = −8.68; p < 0.001.

Regarding the percentage of sperm with normal morphology, we built separate linear regression models for the period 1995–1999 and for the period 2000–2018. In the period 1995–1999 we found a significant tendency towards a reduction in the median value over the years (reduction of 1.9% each year) (Fig. 3A). Controlling for age, we observed a similar tendency towards a decrease in the median percentage of sperm with normal morphology in the three evaluated age groups (reduction of 1.5% per year in the age group <30 years, reduction of 2.0% per year in the age group between 30 and 39 years, and reduction of 1.8% per year in the age group ≥40 years) (Fig. 3B). In the period 2000–2018 we also found a significant tendency towards a reduction in the median value over the years (reduction of 0.53% each year) (Fig. 4A). Controlling for age, we also observed a similar tendency towards a decrease in the median percentage of sperm with normal morphology in the three evaluated age groups (reduction of 0.53% per year in the age group <30 years, reduction of 0.54% per year in the age group between 30 and 39 years, and reduction of 0.54% per year in the age group ≥40 years) (Fig. 4B).

### Sperm concentration after processing

**Figure 5:**
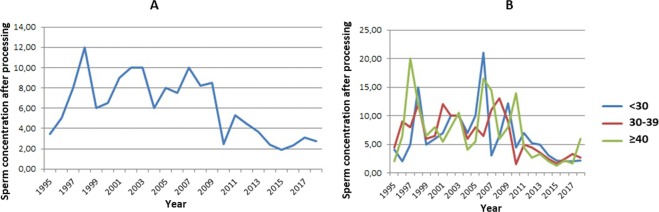
Sperm concentration after seminal processing (millions/ml, median) between 1995 and 2018 - linear regression (n = 3,669). (**A**) – Total group. Total (n = 3,669). Intercept or linear coefficient: A = 8.89; SE(A) = 0.99; t = 9.00; p < 0.001. Inclination or angular coefficient: B = −0.242; SE(B) = 0.074; t = −3.29; p < 0.01. (**B**) – Population categorized by age group. Age < 30 years-old. Intercept or linear coefficient: A = 8.77; SE(A) = 1.79; t = 4.90; p < 0.001. Inclination or angular coefficient: B = −0.194; SE(B) = 0.133; t = −1.46; p = 0.159. Age 30–39 years-old. Intercept or linear coefficient: A = 10.05; SE(A) = 1.16; t = 8.70; p < 0.001. Inclination or angular coefficient: B = −0.301; SE(B) = 0.086; t = −3.49; p < 0.01. Age ≥ 40 years-old. Intercept or linear coefficient: A = 10.53; SE(A) = 1.88; t = 5.61; p < 0.001. Inclination or angular coefficient: B = −0.296; SE(B) = 0.140; t = −2.12; p = 0.04.

Regarding sperm concentration after seminal processing, we found a significant tendency towards a decrease in the median value of post-processing concentration (reduction of 0.24 million/mL each year) (Fig. 5A). Controlling for age, we did not find a downward trend in the median post-processing concentration in men under 30 years of age. In the age group between 30 and 39 years and in the age group ≥40 years, we observed a significant downward trend in sperm concentration after seminal processing (reduction of 0.30 million/mL each year in the two groups) (Fig. 5B).

## Discussion

The theory of declining semen quality in men around the world is still a matter of debate. Our study aimed to investigate the influence of the exam collection year on the semen quality in 9,267 Brazilian men investigated for infertility by analyzing the first semen sample evaluated in our laboratory. The total number of progressive mobile sperm and the percentage of morphologically normal sperm were investigated. In addition, among these samples, 3,669 were also analyzed for sperm concentration after the seminal swim-up processing technique. We observed a reduction in the three parameters analyzed during the period investigated and the results add elements to the discussion about semen quality trends.

According to the standards of the latest WHO manual, to be considered normal, a semen sample should have a sperm concentration ≥15 million/mL and ≥32% of progressively motile sperm^11^. However, some authors suggest that these values have no prognostic relevance^12,13^. In the present study, we analyzed the TMSC, which has been shown to be the parameter with the best correlation with the occurrence of spontaneous pregnancy. According to Hamilton and colleagues, TMSC values greater than 20 × 10^6^ are considered normal^13^.

Between 1995 and 2018, we observed a significant decline in the median TMSC in the three age groups analyzed. Among men aged under 30 years, there was a reduction of 2.67 million/year; among men aged 30–40 years, the reduction was 3.21 million/year; and among men aged over 40 years, we observed a reduction of 2.11 million/year. These results are in line with those recently reported by Tiegs and colleagues. Analyzing the first semen sample of 119,972 men undergoing infertility investigation at two centers, one in Spain and one in the United States, the authors observed that the proportion of men with normal TMSC had dropped by approximately 10% over the past 16 years^14^. Recently, Huang *et al*.^4^ analyzed samples produced by a total of 30,636 young Chinese men who had applied to be sperm donors between 2001 and 2015. Among these, the mean sperm concentration dropped significantly from 68 million/mL between 2001 and 2005 to 47 million/mL between 2011 and 2015. Specifically considering spermatozoa with progressive motility, Huang *et al*. did not find a decreasing trend in relative frequency; however, they observed a significant decrease in the absolute number of spermatozoa, with averages falling from 34 million between 2001 and 2005 to 21 million between 2011 and 2015^4^.

Sperm morphology may influence the chance of spontaneous pregnancy^15^. Authors suggest that the temporal trend of decreasing percentage of sperm with normal morphology can be explained by the different classification criteria used by WHO over the years. The first two editions of the WHO Manual on Human Semen Analysis^16,17^ recommended the use of liberal criteria for morphology classification^18^. The third edition of the manual^19^ recommended that strict criteria should be applied to consider a sperm as morphologically normal and this recommendation was made official in the fourth edition^20^. This made cells previously classified as normal to be considered morphologically altered. We observed a decrease in the median percentage of normal sperm, which went from 17% in 1995 to 4% in 2018. This reduction was significant in the three age groups evaluated, even analyzing the periods 1995–1999 and 2000–2018 separately. It is noteworthy that the median of morphologically normal sperm observed in 2018 is equal to the lower limit of normality according to the latest WHO manual^11^, that is, 50% of men evaluated in 2018 were classified as having teratozoospermia. Similar results were obtained by other authors. In 2017, Huang *et al*. observed a significant reduction in the average number of morphologically normal sperm, which went from 31.8% between 2001 and 2005 to 10.8% between 2011 and 2015^4^. Rolland and colleagues also noted a decrease in normal morphology values, which they believe were not just due to the change in cell classification^21^. Authors who published Brazilian data showed a decline from 4.6% between 2000 and 2002 to 2.7% between 2010 and 2012^8^.

With the advancement of assisted reproduction techniques, there was a need for selection of morphologically normal and non-germ cell-free sperm^11^. The swim-up technique consists of the selection of sperm with greater mobility, ability to swim in the culture medium and theoretically, with greater potential to fertilize the egg^22^. To analyze sperm concentration in the semen after the swim-up technique in 3,669 available samples, we also built different statistical models by age group. Thus, we observed a decrease in median post-processing concentration values in the general group, and more specifically in men over 30 years of age. The median in the overall group which was 8.0 million/mL in 2005 fell to 2.75 million/mL in 2018. This deserves special attention, as some authors consider 5.0 million/mL to be the lowest acceptable value for intrauterine insemination^23^. It is not known to us that other publications have evaluated the temporal tendency of sperm concentration after seminal processing in other populations. We believe this is also a variable that may indicate a decrease in semen quality. This finding is consistent with our finding regarding morphology, as, in theory, the swim-up technique selects morphologically normal sperm^11^.

The possible causes for the fall in some seminal quality parameters are a matter of debate. Smoking^24^, alcohol abuse^25^, lifestyle, excessive coffee consumption^9^, and even the use of mobile phones with radiofrequency electromagnetic radiation emission^26^ are cited as possible culprits of the deterioration of semen quality in recent years. Environmental agents such as air and water pollutants could also act as “endocrine disruptors”. These substances would have estrogen-like effects and could act on the male fetus even during pregnancy, leading to testicular changes known as “testicular dysgenetic syndrome”. This syndrome, in addition to having poor seminal quality, could also increase the frequency of hypospadias, cryptorchidism, and testicular cancer^27^. Among the possible causes for the decrease observed in the seminal quality parameters, one that seems most plausible to us is obesity. In recent years, Brazil has experienced an increase in the prevalence of this disease^28^. Hypoandrogenism due to accumulation of adipose tissue^29^ may impair spermatogenesis, leading to worsening of seminal parameters^30^. Due to the retrospective character of the study, we did not have access to clinical and sociodemographic data of the men who produced the samples, which made it impossible to investigate possible causes for the observed decline in seminal quality.

This study had limitations that should be mentioned. The men evaluated were objectively being investigated for marital infertility. However, the difficulty in obtaining seminal samples is well known^10^, and the population of infertile men is the one that most needs seminal evaluation. Studies with sperm donors may also show the selection bias of men with above average fertility capacity^10^. Over the course of the study, refinements in assisted reproductive technology, including intracytoplasmic sperm injection (ICSI) were developed, which may have influenced men who presented for care. It is known that seminal parameters may vary in the same individual. Since not all men who collected semen in the laboratory produced more than one sample, we chose to analyze each man’s first sample in order to reduce the effects of this possible bias. Although all men received counselling to abstain from sexual activity for 3 to 5 days, we were not able to objectively control this variable. As reported in previous studies, one study period probably should not have been more influenced than the other by this possible confounding factor^31^. We could not establish a hypothesis for the possible decline in seminal quality because we did not have the clinical and sociodemographic data of men. Since semen samples were produced by men residing in a specific region of the country, the conclusions cannot be generalized to other regions of the country and the world. We believe that since we analyzed a considerable number of samples produced over a long period, the results obtained are valid. During the analyzed period there were changes in laboratory techniques that may have influenced the results obtained, however, the fact that the analyzes were always performed in the same laboratory by the same team of professionals reduces the possible interobserver variation.

In conclusion, a possible drop in semen quality could have serious consequences for future generations. From 1995 to 2018, we observed that Brazilian men undergoing marital infertility investigation had a decline in the total number of progressive mobile sperm, a decreased percentage of sperm with normal morphology, and a reduction in sperm concentration after seminal processing. Surveillance of seminal parameters should be maintained in the coming years, and further studies are needed to elucidate possible causes for seminal deterioration and to devise strategies to reverse this trend.

## Methods

### Study population

The Human Reproduction Laboratory (LRH) of University of Campinas (UNICAMP) Women’s Hospital has been conducting sperm analysis since the beginning of its activities, and all results are passed on in a computerized system currently in its latest version. 6.16 of 11/13/2013. This is a system that allows the export of all archived data to Microsoft Excel spreadsheets. This study consists of a retrospective analysis of sperm counts performed between the period 01/03/1995 and 06/29/2018.

For this study, the analyzed population consisted of all male partners of couples admitted to the UNICAMP outpatient clinic for marital infertility investigation. Couples had failed to achieve pregnancy after a period of 12 months or more with regular sex and without any contraceptive method. After exporting all sperm data during this period, a database of semen samples produced by 9,267 men was produced. For the present study, only the first sample produced by each man was analyzed. This database consisted of different sperm parameters besides the date of the exam and the age of the man at the date of the sample collection. There were also data from 3,669 samples of seminal processing performed by the swim-up technique to assess the possibility of future intrauterine insemination. As this was a retrospective analysis, no clinical and sociodemographic data such as race, education, and personal habits were available. All methods were carried out in accordance with relevant guidelines and national regulations. The experimental protocol was approved by the University of Campinas Research Ethics Committee under number 59911516.5.0000.5404. As this was a retrospective study based on database review, not compromising the privacy of subjects, the University of Campinas Research Ethics Committee waived the signing of informed consent.

### Seminal analysis

The men who were going to have the sperm exam were instructed at the time of appointment to maintain sexual abstinence for a period of 3 to 5 days before the date of collection^11^. The collections were performed in a private room, previously sanitized, and in the same laboratory where the analyzes were performed. The seminal sample was obtained by masturbation in a sterile vial previously identified with the patient code. At the end of collection, the vial was delivered by the patient himself to a laboratory professional where he placed it in the incubator at 37 °C for about 1 hour for sample liquefaction. At the end of liquefaction, the sample was transferred to a graduated conical tube with a sterile Pasteur pipette for volume verification. During the whole period of analysis, the samples were taken in the same laboratory and by the same team of biologists with experience in seminal analyzes.

### Evaluation of sperm concentration

Seminal sample dilution was performed in formalin (50 g NAHCO_3_, 10 mL 35% formalin, and distilled water to 1 liter), following the ratio determined by the biologist responsible for the examination. After dilution, an aliquot was placed on either side of the Neubauer chamber. After a period for cell sedimentation, the chamber was analyzed by optical microscope with 10X to 40X magnification, counting the amount of sperm present in the four squares corresponding to the edges, as well as in the central square. Reading was performed in duplicate, and reliability was assessed from WHO standards^11,19,20^. The results of the analysis readings were submitted to a mathematical formula with a correction standard of each dilution to obtain the final concentration of millions/mL.

### Evaluation of sperm motility

A 10 µL aliquot of the semen sample was placed on two coverslips for double evaluation under an optical microscope, following the criteria recommended by WHO manuals^11,19,20^. At least 100 sperm were counted in each slide, classifying them as: grade A (rapid progressive motility or velocity ≥25 µm per second); grade B (slow progressive motility or speed <25 µm per second): grade C (non-progressive motility or speed <5 µm per second); or D (immobile sperm), showing percentage results in each category. Motility was also classified as progressive (sum of percentage of sperm classified as grade A and B) and total motility (sum of percentage of sperm classified as grade A, B, and C)^11^.

### Evaluation of sperm morphology

Simultaneously, two smear slides were prepared, each containing 10 µl of semen, which after drying were subjected to fixation according to the WHO manual^11,19,20^, and after drying, the sperm count was performed. For counting, the slides were placed under 100X magnification with phase contrast. About 100 sperm were analyzed in duplicate and classified as normal or abnormal according to the characteristics of the head, acrosome, intermediate part and tail, plus the size of the sperm and their areas^11,19,20^. Evaluations were performed according to WHO manuals available at the time of the exams.

### Seminal processing

In order to evaluate the possibility of intrauterine insemination, which corresponds to a low complexity assisted reproduction technique, clinicians who performed the couples’ care constantly requested seminal processing with the same sample used for the diagnostic analysis. This was done by two sample washes in a sterile conical tube, adding equal amounts of sperm and commercial PBS (Dulbecco’s phosphate buffered saline; GIBCO Laboratories, Grand Island Biological Co., Grand Island, NY, USA). Two centrifugations were performed in the material for 10 minutes/300 xg each; the supernatant was discarded, and the pellet was stored for the swim-up technique. Next, 1 mL of modified (with HEPES) HTF medium supplemented with 10% Serum Substitute Supplement (SSS_Irvine Scientific, Santa Ana, CA, USA) was added to the remaining pellet and gently homogenized until the pellet dissolved in the medium. This tube containing the material was then tilted at an angle of 45° and incubated at 37 °C for 1 hour. After this period, the first upper mL of the tube was gently removed and transferred to a new sterile conical tube, and 1.5 mL of culture medium was added and reserved for analysis^11,19,20^.

### Evaluation of sperm concentration after seminal processing

An aliquot of the diluted pellet sample was placed in a Makler chamber and analyzed under a 10X to 40X light microscope, counting the sperm present in a row. Reading was performed in duplicate and assessed according to WHO standards^11,19,20^.

### Statistical analysis

The following were considered dependent variables: 1) the total TMSC, corresponding to the volume of ejaculate in mL multiplied by the sperm concentration and the proportion of sperm with motility grade “A” and “B” divided by 100, 2) the percentage of sperm with normal morphology, 3) the sperm concentration in semen after seminal processing. The independent variable was the year of semen collection. The age of the man at the time of collection was considered as a control variable. Initially, a descriptive analysis of each dependent variable was performed, with calculation of mean, standard deviation, median, and 5th, 25th, 75^th^, and 95th percentiles. To evaluate the dependent variables over the years of collection, a linear regression analysis for the median values was used, due to the absence of normal distribution, both in the general population and in the population categorized by age group (<30, 30–39, and ≥40 years). The classification criteria of sperm morphology have been modified over the years. In the period from 1995 to 1999, the WHO laboratory manual for the examination and processing of human semen recommended that strict criteria for classification of sperm morphology should be used in place of the liberal criteria that had been previously used^19^. In the manual published in 1999, WHO made this recommendation official^20^. To avoid bias in the analysis of this variable, we built separate linear regression models for the period 1995–1999 and for the period 2000–2018, both in the general population and in the population categorized by age group (<30, 30–39, and ≥40 years). The significance level was set at 5%, and the program used for the analysis was SPSS version 20 (IBM Corp., Armonk, NY, USA).

## Data Availability

The datasets generated during and/or analyzed during the current study are available from the corresponding author on reasonable request.
